# Design and Feasibility of a Text Messaging Intervention to Prevent Indoor Tanning Among Young Adult Women: A Pilot Study

**DOI:** 10.2196/mhealth.6493

**Published:** 2016-12-22

**Authors:** William D Evans, Darren Mays

**Affiliations:** ^1^ Department of Prevention and Community Health Milken Institute School of Public Health The George Washington University Washington, DC United States; ^2^ Lombardi Comprehensive Cancer Center Georgetown University Medical Center Georgetown University Washington, DC United States

**Keywords:** indoor tanning, risk perceptions, text messaging, feasibility testing

## Abstract

**Background:**

Although skin cancer is largely preventable, it affects nearly 1 of 5 US adults. There is a need for research on how to optimally design persuasive public health indoor tanning prevention messages.

**Objective:**

The objective of our study was to examine whether framed messages on indoor tanning behavioral intentions delivered through short message service (SMS) text messaging would produce (1) positive responses to the messages, including message receptivity and emotional response; (2) indoor tanning efficacy beliefs, including response efficacy and self-efficacy; and (3) indoor tanning risk beliefs.

**Methods:**

We conducted a pilot study of indoor tanning prevention messages delivered via mobile phone text messaging in a sample of 21 young adult women who indoor tan. Participants completed baseline measures, were randomly assigned to receive gain-, loss-, or balanced-framed text messages, and completed postexposure outcome measures on indoor tanning cognitions and behaviors. Participants received daily mobile phone indoor tanning prevention text messages for 1 week and completed the same postexposure measures as at baseline.

**Results:**

Over the 1-week period there were trends or significant changes after receipt of the text messages, including increased perceived susceptibility (*P*<.001), response efficacy beliefs (*P*<.001), and message receptivity (*P*=.03). Ordinary least squares stepwise linear regression models showed an effect of text message exposure on self-efficacy to quit indoor tanning (*t*_6_=–2.475, *P*<.02). Ordinary least squares linear regression including all measured scales showed a marginal effect of SMS texts on self-efficacy (*t*_20_=1.905, *P*=.08). Participants endorsed highly favorable views toward the text messaging protocol.

**Conclusions:**

This study supports this use of mobile text messaging as an indoor tanning prevention strategy. Given the nature of skin cancer risk perceptions, the addition of multimedia messaging service is another area of potential innovation for disseminating indoor tanning prevention messages.

## Introduction

Although skin cancer is largely preventable by reducing ultraviolet radiation exposure, the incidence has increased [1,2], affecting nearly 1 of 5 US adults and incurring significant costs to society [3-5]. Indoor tanning increases the risks of skin cancer [6,7], causing an estimated 10% of all cases [8]. Among US adults, indoor tanning is most prevalent among white (Hispanic and non-Hispanic) young adult women aged 18 to 30 years, with up to 30% of this group tanning each year [9-11]. Indoor tanning among young women further increases skin cancer risks [6,7,12,13] and leads to early-onset disease [14].

The 2014 *Surgeon General’s Call to Action to Prevent Skin Cancer* called for research on how to optimally design persuasive public health indoor tanning prevention messages targeting young women [5]. In other areas of cancer prevention and control (eg, tobacco control), public health communication messaging is a recommended best practice for preventing and reducing behavioral risk factors for cancer [15,16]. Such messaging approaches are designed for wide reach and impact because they can be delivered through channels such as the Internet, mobile phones, and public health campaigns [15,16]. In settings such as Australia, public health messaging campaigns have helped to curb a growing skin cancer epidemic [17]. However, research conducted to date on skin cancer prevention messaging has focused primarily on sun safety behaviors (eg, sunscreen use) [18,19]. The available evidence on indoor tanning behavior among young women suggests different motives may drive intentional indoor tanning behaviors (eg, improving physical appearance) and override perceived short- and long-term risks [5,20-22] (also DM and WDE, unpublished data, 2016).

The extended parallel process model provides a theoretical perspective for how to design and frame persuasive indoor tanning prevention messages [23]. However, evidence of the effects of persuasive public health messages that attempt to frame the potential benefits (gain) and consequences (loss) of skin cancer preventive behaviors is mixed. Some studies favor gain-framed messages [24] emphasizing the benefits of behaviors such as sun protection, while some favor loss-framed messages conveying potential risks [25]. Other studies show no distinct advantage of either gain- or loss-framed messages [26]. Meta-analyses generally reflect these mixed results [27], with no clear advantage of either message frame emerging for sun protection behaviors. However, prior messaging studies have focused primarily on sun protection behaviors (eg, seeking shade, using sunscreen), and research investigating the effects of persuasive, framed messages for preventing indoor tanning is scarce [28,29].

Research on how to craft effective indoor tanning prevention messaging in such a way that addresses the unique motives of this behavior among young women is needed for national skin cancer prevention efforts. Research is needed not only on the *content* and *framing* of persuasive indoor tanning prevention messaging, but also on the optimal *delivery modality.*

Mobile devices are virtually ubiquitous among US young adults: 85% of US young adults own a smartphone with multimedia and Internet capabilities, and virtually all of these young adults use their device to some extent for short message service (SMS) text messaging [30]. Mobile devices are also a popular medium for delivering behavior change interventions, medication reminders, treatment information, and adherence tools targeted to improve health outcomes [31]. Research in this area indicates mobile text messaging interventions are effective for promoting healthy behavior change, including weight loss [32], nutrition and physical activity [33,34], smoking cessation [31,35,36], and prenatal behaviors among pregnant women [37,38].

These examples in the literature, and the widespread use of mobile phone text messaging among young adults, provide support for mobile phone text messaging as a medium for delivering persuasive messaging to prevent indoor tanning among young women. However, few previous studies in this area have harnessed the potential of optimally framed messages delivered via text message.

We designed this study to build from previous research and inform indoor tanning prevention efforts targeting young adult women by examining the effects of gain-, loss-, and balanced-framed text messages on indoor tanning behavioral intentions. The overall hypothesis was that delivering indoor tanning prevention messages both on the Web and via mobile phone text messages to young adult women who indoor tan would be feasible and that procedures would be acceptable to study participants. Specifically, this study explored 3 research questions (RQs): (1) Do participants respond positively to the text messages, as measured by message receptivity and emotional response? (2) Is receipt of text messages associated with changes in indoor tanning efficacy beliefs, including response efficacy (ie, perceived benefits of avoiding indoor tanning) and self-efficacy to avoid tanning? (3) Is receipt of text messages associated with changes in indoor tanning risk beliefs, including perceived severity and perceived personal susceptibility to the risks? We examined these questions in a pilot study to evaluate the feasibility of using SMS texts as a delivery modality for indoor tanning prevention messages. Additionally, we collected qualitative feedback from participants on their perceptions of the indoor tanning prevention messages. We report on these data to help interpret our quantitative pilot results.

## Methods

### Protocol

We conducted a pilot study to test the feasibility and acceptability of delivering persuasive gain-, loss-, and balanced-framed messages on the Web and via mobile phone text messaging in a sample of 21 young adult women who indoor tan (mean age 24.9 years, SD 2.9; 9/21, 43% frequent tanners). The pilot drew from our previous experience in similar studies of persuasive messaging for cancer prevention and control, and other health behavior domains [39,40] (also DM and WDE, unpublished data, 2016). Study inclusion criteria were as follows: (1) white females 18-30 years of age, (2) having had 1 or more indoor tanning exposures in the past 12 months, (3) willing to send and receive text messages via a personal mobile phone, and (4) able to complete all study assessments and procedures in English.

Potential participants were young adult women who had participated previously in an observational study on indoor tanning behavior [41] and met study eligibility criteria. Participants were initially contacted by email with a brief description of study procedures and an invitation to participate, and those who contacted study personnel expressing interest were rescreened for eligibility for the study by telephone. Eligible participants then received informed consent forms, and enrollment was complete once informed consent forms were signed and returned to study personnel. Nearly all participants screened for the pilot (20/21, 95%) were eligible, consented to participate, and completed study procedures.

Eligible, consenting participants completed baseline measures through a custom website and at the conclusion of the baseline assessment were randomly assigned to receive gain-, loss-, or balanced-framed text messages. Participants were randomly assigned in a 1:1:1 ratio to these conditions using an algorithm embedded within the Web-based survey software (Qualtrics Research Suite, Qualtrics LLC). Participants then completed immediate postexposure measures of behavioral intentions, indoor tanning risk (perceived severity, susceptibility) and efficacy (self-efficacy, response efficacy) beliefs, and message response (emotional response, message receptivity). Then for a 1-week period participants received daily mobile phone indoor tanning prevention text messages. At study enrollment, we assessed participants’ preferred time of message delivery relative to the typical time of day they indoor tan (morning, afternoon, evening) and scheduled message delivery accordingly. Participants responded to an initial message reporting whether they indoor tanned that day. We tailored 2 subsequent messages to their reported tanning behavior each day, as the examples in Table 1 show. The initial message delivered fact-based information on indoor tanning risks and the benefits of avoiding indoor tanning. The second message served as a prompt to encourage behavior change (avoiding indoor tanning) or to maintain indoor tanning avoidance [42]. The tailoring algorithm and delivery schedule was preprogrammed into a commercially available messaging software system (TextIt, Nyaruka Ltd). After receiving messages for 1 week, participants completed a Web-based follow-up with measures similar to the baseline assessment.

All participants provided written informed consent, and study procedures were reviewed and approved by the Georgetown University Institutional Review Board.

**Table 1:** Examples of initial (SMS1^a^) and follow-up prompt (SMS2) indoor tanning prevention text messages for day 1 (of 7 days), by message framing condition (gain-, loss-, or balanced-framed), based on whether a participant answered “yes” or “no” to whether they had indoor tanned that day.

Text message	Text message condition						
	Gain		Loss		Balanced	
	Yes, tanned	No, did not tan	Yes, tanned	No, did not tan	Yes, tanned	No, did not tan
SMS1, 160-character maximum	Every time you tan, you’re damaging the DNA in your skin cells. Keep your skin safe and healthy. Avoid indoor tanning to prevent skin damage.	Every time you tan, you’re damaging the DNA in your skin cells. Keep your skin safe and healthy. Avoid indoor tanning to prevent skin damage.	Every time you tan, you’re damaging the DNA in your skin cells. Indoor tanning causes skin damage.	Every time you tan, you’re damaging the DNA in your skin cells. Indoor tanning causes skin damage.	Every time you tan, you’re damaging the DNA in your skin cells. Avoid indoor tanning to prevent skin damage.	Every time you tan, you’re damaging the DNA in your skin cells. Avoid indoor tanning to prevent skin damage.
SMS2	Quitting indoor tanning is easy! Quitting indoor tanning now will keep your skin safe and healthy.	Avoiding indoor tanning is easy! Keep avoiding indoor tanning so your skin stays safe and healthy.	Quit indoor tanning now to avoid skin damage.	Avoid indoor tanning so you don’t damage your skin.	Quitting indoor tanning is easy! You can quit indoor tanning now to keep your skin safe and healthy.	Avoiding indoor tanning is easy! Keep avoiding indoor tanning so your skin stays safe and healthy.

^a^SMS: short message service.

### Preexposure Measures

Prior to any message exposure, we captured demographic characteristics (age, household income, whether participants were current students) and past year indoor tanning behavior using validated items from epidemiological surveys [10]. To characterize the sample, we operationalized frequent indoor tanning based on a binary variable indicating indoor tanning 10 or more times in the past year [10].

### Text Messages

We developed a 7-day program of text messages to be delivered to participants after we collected baseline data. Messages were designed based on the lead author’s previous studies on cancer prevention messages and SMS for young women [38,39]. We developed message content based on indoor tanning prevention messages we previously tested [28,29], skin cancer prevention text messaging research [42], and research on indoor tanning beliefs, motives, and health risks [5,14,43]. Content was tailored to participants’ reported indoor tanning behavior each day and was framed based on the conditions to which participants were randomly assigned. Table 1 provides examples of the gain, loss, and balanced text messages delivered on day 1 of the pilot. Note that we first sent an initial message to get the participant’s attention, followed by a prompt message framed as gain, loss, or balanced.

### Postexposure Measures

We administered the following measures immediately after participants were exposed to the Web-based messages at baseline, and at the conclusion of the 1-week text message exposure period to capture participants’ responses to the indoor tanning prevention messages.

#### Emotional Response

Emotional response to the messages was measured with 3 items from prior research assessing whether participants felt frightened, anxious, or nervous while reading the message [44]. Responses were based on a 4-point scale (1=not at all, 4=extremely) and were averaged to create a score, with higher values indicating stronger fear responses (Cronbach alpha=.89 at both time points).

#### Message Receptivity

Receptivity to the messages was measured using an adapted 7-item scale [28]. Examples of items are “The message was convincing,” “The message said something important to me,” and “The message gave me a good reason not to tan indoors.” Participants responded to the statements on a 7-point scale (1=strongly disagree, 7=strongly agree), and responses were averaged to create a score, with higher values indicating greater message receptivity (Cronbach alpha range .79-.82).

#### Risk Beliefs

Perceived severity of the risks of skin cancer was measured using 5 items adapted from previous research [45] with a 5-point response scale (1=strongly disagree, 5=strongly agree). The items were averaged to create a score, with higher values indicating greater perceived severity (Cronbach alpha range .70-.73). Perceived susceptibility to skin cancer was measured using 6 items adapted from a previous study [45]. Responses were based on a 5-point scale (1=strongly disagree, 5=strongly agree) and were averaged to create a score, with higher values indicating greater perceived susceptibility (Cronbach alpha range .80-.81).

#### Efficacy Beliefs

Response efficacy was assessed using a 7-item scale adapted from other cancer prevention risk behavior research to capture the perceived health benefits of avoiding indoor tanning (eg, reducing risks of skin cancer) [28,37]. Responses were based on a 9-point scale (1=no chance, 7=certain to happen) and averaged to create a summery score, with higher values indicating stronger perceived response efficacy (Cronbach alpha range .81-87). Self-efficacy was measured using 2 items assessing how confident participants were and how easy it would be for participants to quit indoor tanning in the next year [28]. Responses were based on a 7-point scale (1=not at all, 7=extremely) and averaged to create a summary score, with higher values indicating greater self-efficacy (Cronbach alpha range .75-.85).

#### Indoor Tanning Behavioral Intentions

Similar to previous studies [28,29], the primary outcome measured following exposure to the messages at baseline and the 1-week posttest was indoor tanning behavioral intentions. We chose this outcome because behavioral intentions have been demonstrated in previous research to predict future health behavior change [46] and could be assessed as a potential indicator of future behavior change in a pilot study with limited follow-up duration. For this study, we captured behavioral intentions to indoor tan using 2 items assessing intentions to tan even once and intentions to tan regularly in the next year on a 7-point scale (1=definitely will not, 7=definitely will) [28]. Intentions to quit indoor tanning were measured using a single item assessing how much the message made participants want to avoid indoor tanning in the next year on a 7-point response scale (1=not at all, 7=a lot) [28,29]. These items were moderately correlated (*r* range .51-.76) and had good internal consistency when we reverse coded the intentions-to-quit item and we considered items as a single behavioral intentions construct (Cronbach alpha range .73-.84). We averaged the items to create a summary variable, where higher values indicated stronger behavioral intentions to indoor tan.

At the 1-week posttest, we also administered items assessing the acceptability of the study procedures and willingness to participate in such studies in the future [47]. Specifically, these measures captured whether participants found completing study procedures to be easy, if they encountered any challenges, and how long they would be willing to participate in such a study in the future.

### Data Analysis

All data analyses were performed with IBM SPSS version 19 (IBM Corporation). We examined the variables of interest descriptively to characterize the sample, and used bivariate analyses to test for differences across the experimental conditions. No participant characteristics differed by study condition; therefore, we did not adjust analyses for covariates. To examine changes over the 1-week exposure period in variables of interest, we used paired *t* tests to compare means at baseline and 1 week posttest. To evaluate the effects of the texts on the dependent measure of intentions to indoor tan, and due to the small sample size, we estimated 2 ordinary least squares (OLS) linear regression models. First, we used a stepwise procedure [48]. In this OLS analysis, the posttest measures of risk and efficacy beliefs and message response were regressed onto the dependent variable of indoor tanning behavioral intentions at posttest. Second, we estimated an OLS model using all posttest measures regressed onto the indoor tanning intentions variable at posttest. As noted above, in all analyses the variable for indoor tanning behavioral intentions was an average of 3 items, with higher values indicating stronger intentions to tan on a 1 to 7 scale.

## Results

### Descriptive Results

We achieved 100% (21/21) compliance with the daily text messaging protocol, with all participants sending and receiving daily text messages. Table 2 shows a comparison of measures administered after exposure to Web-based messages at baseline and after the text messaging exposure at 1 week. Over this brief period, we observed trends or significant changes in the variables of interest after receipt of the tailored text messages for 1 week, including increased perceived susceptibility (*P*<.001), response efficacy beliefs (*P*<.001), and message receptivity (*P*=.03).

### Multivariate Regression Models

Next, we analyzed the data using an OLS linear regression with a stepwise algorithm. One indoor tanning belief scale, self-efficacy to quit indoor tanning, emerged as statistically significant and was positively correlated with text message exposure at follow-up. Table 3 and Table 4 summarize these results.

**Table 2:** Comparison of measures at baseline and 1 week (N=21).

Measures	Baseline	After 1 week	*P* value
	Mean (SD)	Mean (SD)	
Perceived susceptibility	3.38 (0.844)	3.84 0 (.746)	<.001
Perceived severity	3.89 (0.736)	4.06 (0.608)	.24
Self-efficacy	5.43 (1.39)	5.45 (1.52)	.64
Response efficacy	4.98 (0.743)	5.65 (0.881)	<.001
Emotional response	2.11 (0.845)	1.98 (0.792)	.31
Message receptivity	5.46 (1.04)	5.80 (0.812)	.03
indoor tanning behavioral intentions	3.30 (1.34)	2.92 (1.39)	.11

**Table 3:** Ordinary least squares stepwise linear regression of indoor tanning behavioral intentions on beliefs.

Model	B	SE	Beta	*t* ^a^	*P* value
Constant	5.382	1.031		5.222	<.001
Self-efficacy to quit indoor tanning	–.451	0.182	–.494	–2.475	.02

^a^*df*=6 for the regression; *df*=14 for the residual; *df*=20 for the total model.

**Table 4:** Excluded variables in stepwise procedure.

Variables	Collinearity statistics				
	Beta	*t* ^a^	*P* value	Partial correlation	Tolerance
Risk beliefs	–.147	–0.725	.48	–0.168	0.991
Perceived severity	–.158	–0.762	.46	–0.177	0.944
Response efficacy	–.052	–0.232	.82	–0.055	0.828
Emotional response	–.272	–1.343	.20	–0.302	0.93
Message receptivity	–.344	–1.777	.09	–0.386	0.957

^a^*df*=6 for the regression; *df*=14 for the residual; *df*=20 for the total model.

**Table 5:** Ordinary least squares linear regression with all variables included (no stepwise procedure).

Variables	Unstandardized coefficients			Standardized coefficients		
	B	SE	Beta	*t* ^a^	*P* value
(Constant)	8.423	2.641		3.1899	.007
Risk beliefs	0.109	0.517	.0599	0.2111	.84
Perceived severity	0.051	0.664	.0222	0.0777	.94
Self-efficacy	–0.432	0.227	–.472	1.905	.08
Response efficacy	–0.018	0.492	–.012	–0.037	.97
Emotional response	–0.316	0.485	–0.181	–0.651	.53
Message receptivity	–0.525	0.458	–0.308	–1.146	.27

^a^*df*=6 for the regression; *df*=14 for the residual; *df*=20 for the total model.

Next we ran the OLS linear regression without the stepwise algorithm, including all indoor tanning belief and message response predictor variables, summarized in Table 5. In this model, self-efficacy was no longer significantly associated with indoor tanning behavioral intentions at *P*>.05, but this result was near significance at *P*=.08.

### Text Message Acceptability

Finally, we asked participants follow-up questions regarding the text messaging and their participation in the pilot study. In response to these questions, respondents all indicated that it was easy to participate in the pilot (21/21) and that they did not find it challenging or difficult (21/21); 81% of participants (17/21) indicated that they would be willing to participate in a future text messaging study related to indoor tanning for a period of 4 weeks. These results generally indicate the intervention was well received and feasible for participants, suggesting the opportunity to conduct additional text messaging for indoor tanning prevention studies in the future.

## Discussion

### Principal Findings

Overall, regarding RQ1, we found that respondents were receptive and responded positively to the text messages. Our descriptive results showed higher receptivity using the validated message receptivity scale at 1-week follow-up, based on our descriptive results. For RQ2, descriptive results showed both that self-efficacy (to avoid indoor tanning) and response efficacy (perceiving that avoiding tanning will be beneficial) increased after text message exposure. Results from the OLS models were mixed, with self-efficacy increasing in the stepwise procedure, but not reaching significance in the full model. Our limited sample size and the pilot nature of the research should be considered in interpreting these findings. Finally, for RQ3, descriptive results showed that perceived severity and perceived personal susceptibility both increased after exposure to the text. However, the OLS models revealed no effects on these risk beliefs.

Regarding our overall goal to assess feasibility of using SMS for indoor tanning prevention, we found that text messaging was an acceptable approach to delivering indoor tanning prevention messages. We achieved total compliance with the intervention in the pilot sample, and participants expressed high levels of satisfaction and ease of use. These results are consistent with previous SMS interventions but are novel results for the subject matter of tanning prevention [34,49]. Self-efficacy appears to be an important belief to promote, as it was associated with higher intentions to avoid future indoor tanning in our sample. These results are novel and suggest that SMS is a promising approach for preventing indoor tanning.

Text messaging is a proven intervention strategy in several domains of public health [49]. While text messaging has long been used for treatment adherence and as a reminder system, there is now solid evidence of texting for behavior change, especially in smoking cessation and antiretroviral therapy promotion [31], and it is growing in popularity in other domains [50]. Texting interventions have been shown to be effective in changing specific targeted behaviors through persuasive messaging [38].

However, there has been virtually no previous work on text messaging for indoor tanning prevention [51]. Given the widespread use of mobile phone text messaging among young adults, using mobile phone text messaging as a medium for delivering persuasive messaging to prevent indoor tanning among young women, the population subgroup where indoor tanning is most prevalent, is especially promising. This study provides initial evidence that indoor tanning prevention text messaging is feasible, is accepted by the at-risk target population of young women, and shows short-term effects of exposure to texting on indoor tanning behavioral intentions and other indoor tanning belief measures. This suggests that text messaging can potentially be implemented in a longer intervention period in the future.

Future research on indoor tanning messaging should include more measurement and analysis of dosage, and other means of optimizing message delivery. For example, studies have examined point-of-decision prompts to increase exercise and nutrition [52] and use of mobile technologies for health interventions [53], but, to our knowledge, no study has combined both. Text messaging interventions can do much more than simply deliver text reminders—they can deliver, right into the hands of a highly targeted population, the public health messages that in years past would have appeared in mass media [54]. There is potential to extend these areas of future research to indoor tanning messaging.

Future research should consider the nature of skin cancer risk perceptions and explore the addition of multimedia messaging service (MMS) delivery. MMS is another area of potential innovation that may enhance the persuasive appeal of indoor tanning prevention messages. The full capabilities of mobile technology using graphic imagery, video, and other multimedia via MMS to deliver persuasive indoor tanning prevention messages along with text-based content should be explored. MMS also allows for interactive communications, content tailoring, and embedding of photos and images into text messages delivered to mobile devices. These features appear promising for indoor tanning prevention messaging given the importance of skin appearance, especially to priority populations at risk from indoor tanning exposure, such as young adult women.

### Study Limitations

Our results should be interpreted in light of two important limitations. First, this was a pilot study with a small convenience sample, and results were evaluated over a short period of 1 week of text messaging exposure. Thus, we did not evaluate the long-term effects of texting exposure and known variables that have affected results of previous mHealth trials, such as dosage, potential “wear-out” effects of long-term message exposure, timing and sequencing, and message content. Second, we did not examine sociodemographic and other subgroup differences due to the small sample size. Some of these variables, such as age and race/ethnicity, have been found to affect results of previous indoor tanning prevention interventions [28,29]. These factors should be part of future studies with larger samples conducted over extended time periods.

### Conclusions

Overall, this study provides preliminary evidence for the effectiveness of SMS in promoting indoor tanning prevention beliefs and for increasing risk beliefs concerning the health consequence of tanning. It also shows that an SMS intervention is acceptable and may be a feasible health communication channel for indoor tanning prevention. Based on our results, text messaging appears to be a way in which persuasive messages may be tailored to the at-risk population of young adult women and to an optimal time for delivery [55].

## Abbreviations

MMS: multimedia messaging service
OLS: ordinary least squares
RQ: research question
SMS: short message service
